# Quality of emergency rooms and urgent care services: user satisfaction

**DOI:** 10.1590/S1679-45082015GS3347

**Published:** 2015

**Authors:** Cássio de Almeida Lima, Bruna Tatiane Prates dos Santos, Dina Luciana Batista Andrade, Francielle Alves Barbosa, Fernanda Marques da Costa, Jair Almeida Carneiro

**Affiliations:** 1 Universidade Estadual de Montes Claros, Montes Claros, MG, Brazil.; 2 Faculdade de Saúde Ibituruna, Montes Claros, MG, Brazil.

**Keywords:** Patient satisfaction, Patient care, Quality of health care, Health evaluation, Emergency medical services, Questionnaires

## Abstract

**Objective:**

To evaluate the quality of emergency rooms and urgent care services according to the satisfaction of their users.

**Methods:**

A cross-sectional descriptive study with a quantitative approach. The sample comprised 136 users and was drawn at random. Data collection took place between October and November 2012 using a structured questionnaire.

**Results:**

Participants were mostly male (64.7%) aged less than 30 years (55.8%), and the predominant level of education was high school (54.4%). Among the items evaluated, those that were statistically associated with levels of satisfaction with care were waiting time, confidence in the service, model of care, and the reason for seeking care related to acute complaints, cleanliness, and comfortable environment.

**Conclusion:**

Accessibility, hospitality, and infrastructure were considered more relevant factors for patient satisfaction than the cure itself.

## INTRODUCTION

The worldwide phenomenon of overcrowding that occurs in hospital emergency services is also a Brazilian reality. Several factors are involved in the genesis of this problem, and the most expressive are increased urban violence, greater incidence of problems with external causes, and aging population, which influence the increase in prevalence of chronic degenerative diseases. This demand for emergency hospital services implies delays in care and an overworked healthcare team, leading to low quality care.^1,2^

This difficulty in the urgency and emergency healthcare services exists from the level of Primary Care, due to the scarcity of specialized professionals, in parallel with the low rate of resolution and the reduced health promotion strategies, which lead the user to seek the emergency room that is open 24-hours a day. Therefore the emergency room receives a high demand of patients, which interferes in the care given to the clientele that needs real emergency care, and hinders those sent from the primary care centers, making the client go through refusals, long waiting lines, sluggishness, and delay in being seen.^3,4^

Despite the processes in evolution within the Unified Healthcare System (*Sistema *Único* de Saúde* - SUS), for the national health system, the constitutional principles should be respected and pursued. Likewise, the various aspects that compromise hospital care, such as those related to human resources and the organization of the Healthcare Networks, which should be integrated, deserve to be the object of evaluations and effective interventions.^5^ In this scenario, the assessment of care from the user’s viewpoint becomes extremely relevant, that is, the user’s satisfaction as to the quality of this care. In Brazil, user satisfaction surveys have received special attention as of the 1990’s, due to re-democratization of the country and the appearance of movements for social rights, especially those of access to healthcare services.^2^

In this way, the user is given the opportunity to give opinion about the public policies and identify the determining factors of his/her satisfaction, along with the recommendation of the World Health Organization (WHO) that managers take into consideration the expectations of the citizens in decision-making processes. For this, patient satisfaction has been adopted by healthcare institutions as a strategy for obtaining a set of perceptions related to the quality of care received, with which one acquires information that will benefit the service organization.^6^

Since then, literature has become very ample on this theme. However, more specific studies are needed that point out aspects directly related to the quality of the service and the satisfaction of the users, underlining the factors related to the client, the healthcare professionals, and to the quality of the facilities.^10^Since the goal of patient care is the patient, it is more than justified to seek quality in their expectations, aiming at their satisfaction as a determining aspect for judging quality.

In this sense, in search of a better quality of healthcare in which urgency and emergency services stand out, an increased quantity of evaluations by the organizations is observed at national and international levels. This is because researching on patients’ satisfaction makes possible their participation in the evaluation and decision-making process, which promotes effective and appropriate improvements for the clientele seen. These are in agreement with the current WHO recommendations and the assumptions of social participation of the SUS. Thus, it is believed that this study should guarantee useful information to managers and professionals who deliver care in urgency and emergency services, besides subsidizing more discussions on the subject.

## OBJECTIVE

To evaluate the quality of the emergency rooms and urgent care services according to satisfaction of their users.

## METHODS

This was a cross-sectional descriptive study, with a quantitative approach. The following hospitals from the city of Montes Claros (MG) were included: *Hospital Universitário Clemente de Faria *(HUCF),* Fundação Hospitalar de Montes Claros – Hospital Aroldo Tourinho *(HAT),* Irmandade Nossa Senhora das Mercês de Montes Claros (Santa Casa), *and* Fundação de Saúde Dilson de Quadros Godinho *(FDQG).

The focus of the study was the emergency rooms and urgent care services of these hospitals which represented the hospital universe of the city of Montes Claros (MG). We point out that all these services were the entrance way for users, with no requirement for a recommendation in order to be seen. It is worth mentioning that some particularities of the services, namely: only the HUCF was an emergency room that cares exclusively for patients from the SUS. In this emergency room, there were 36 beds for observation or intermediate care service for those who awaited hospital admission. For each 12-hour work shift, the service counted on one internal medicine physician, one pediatrician, one surgeon, and one orthopedic surgeon, besides two nurses, one of them responsible for triage and the other, for care; it also had six nurse technicians. The other organizations render services to patients from the SUS, from health insurance companies and private patients.

HAT, *Santa Casa*, and HUCF had emergency rooms open for 24 hours. FDQG only had an urgent care service. The emergency room at HAT had 25 beds; for each 12-hour work shift, there was one internal medicine physician, one pediatrician, one surgeon, one orthopedic surgeon, and one neurologist, besides two nurses and four nurse technicians. At the *Santa Casa*, the emergency room had 40 beds and had the following team: two internal medicine physicians, two pediatricians, one surgeon, one orthopedic surgeon, one cardiologist, and one neurologist, in addition to two nurses and eight nurse technicians. As to FDQG, in the urgent care service, there were 12 beds, besides a staff team composed of one internal medicine physician, one pediatrician, one surgeon, two nurses, and three nurse technicians.

To define the size of the sample, the 20% cut-off point was used for all the subjects seen in one day of normal service. The basis for calculation was the number of daily cases recorded by means of the outpatient information system, that is, the daily mean of cases seen. At the time of data collection, the services daily cared for the following average numbers of patients: HUCF − 100 patients; HAT − 100 patients; *Santa Casa* − 300 patients; FDQG − 180 patients. The total was 680 patients/day, on average.

Considering 20% of the total, the sample was composed of 136 users. The choice of 20% of the population was justified by the fact of its being an infinite and fluctuating population, for which a proportion needed to be defined and could vary between 10 and 50%. However, since it was a commonplace and recurring phenomenon – care delivered in health services – the 20% proportion was sufficient to define and characterize the phenomenon.^14^

Random and stratified sampling was considered. The extracts of the sampling process were each one of the emergency rooms or urgent care services. Considering that each healthcare service in the study was an extract, the due proportions were respected of the number of cases seen/day at each service, investigating different quantities of users in each service relative to the total of 136 patients/day in emergency rooms and urgent care services in Montes Claros (MG). They were divided as follows: HUCF with 14.7% (20 individuals); HAT with 14.7% (20 individuals); *Santa Casa* with 44.1% (60 individuals), and FDQG with 26.4% (36 individuals).

Study participant selection occurred by means of a drawing using the lists of people who had their risk classified by the Manchester protocol and who were awaiting a return visit and discharge after medical care. Included in the study were the users who had already been seen and awaited discharge and who did not present with any condition that would limit their participation in the study at the time of the interview, such as intense pain, or any other significant discomfort; were aged 18 years or over; and agreed to answer the questionnaire after previously reading and signing the Informed Consent Form.

Data collection was performed between October and November, 2012, by means of a questionnaire applied to the selected users. During the application of the questionnaires, the clients who were called to their clinical visit had their interview interrupted, in order to not delay care; these were automatically eliminated from the sample. In this case, the participant was randomly substituted by another. The questionnaires were always dealt with during the day shift.

The questionnaire used to collect data was structured and validated by the Ministry of Health, by means of the National Program of Hospital Service Evaluation.^15^ Other questions were included about knowledge of the users as to what urgent and emergency care were, and how the healthcare network should be organized.

The independent variables of the study were grouped into sociodemographics, characteristics of care at the urgent and emergency care services, and characteristics of the facilities. The sociodemographic characteristics included age, gender, and level of schooling. The characteristics of care variables were waiting time, confidence in the service, whether patients received explanations about their health status, respect of the medical and nursing teams, in addition to the name of the professional who cared for that patient, concept of risk of one’s own health, whether before coming to the emergency room the patient sought any other primary care center, and reason for seeking the service. The characteristics of the facilities variables were appropriate cleanliness, quality, and comfort of the facilities, if the user knew where to complain in case of dissatisfaction. The dependent variable was defined by the following question: “Are you satisfied with the care you received at the emergency room or urgent care service?” The “satisfied” category included those who reported feeling very satisfied and satisfied, and the unsatisfied or very unsatisfied were included in the “unsatisfied” category.

The statistical analyses were done using the software Statistical Package for Social Sciences (SPSS), version 18.0 for Windows. After the descriptive analysis, the association between the report of satisfaction with care delivered and the independent variables was investigated by means of bivariate analysis, using the Pearson χ^2^ test. For all the analyses, p≤0.05 was considered.

The research project was approved by the Research Ethics Committee of the *Faculdades Unidas do Norte de Minas - Associação Educativa do Brazil,* with the official document 225,868/2013 and CAAE: 06316712.1.0000.5141.

## RESULTS

A total of 136 users of the urgent care and emergency room services participated in the study. The results showed a great dissatisfaction with the care given in the emergency rooms and urgent care services of the hospitals studied: 94 (69.1%) of users were dissatisfied. The sample comprised patients who were, in the majority, females (88; 64.7%) aged up to 30 years (76; 55.9%), and the predominant level of schooling was high school (74; 54.4%).

The factors associated with satisfaction and dissatisfaction in the bivariate analysis (p≤0.05) were delay in being seen, waiting time, confidence in the service, opinion on the model of care, and reason for seeking the emergency room (Table 1).

**Table 1:** Result of the bivariate analysis between satisfaction/dissatisfaction, and the variables relative to the user profile and characteristics of care (n=136)

Variables	Satisfied n (%)	Dissatisfied n (%)	p value*
Sex			
Female	16 (11.8)	32 (23.5)	0.40
Male	26 (19.1)	62 (45.5)	
Age			
Less than 30 years	22 (16.2)	54 (39.7)	0.35
More than 30 years	20 (14.7)	40 (29.4)	
Level of schooling			
Higher education	7 (5.1)	12 (8.8)	0.35
High school	19 (14.0)	55 (40.4)	
Illiterate to high school	16 (11.8)	27 (20.0)	
Characteristics of care delivered			
Delay in care			0.00
No delay	6 (4.4)	0 (0)	
Delayed	36 (26.5)	94 (69.1)	
Waiting time			0.00
Up to 120 minutes	40 (29.4)	54 (39.7)	
More than 120 minutes	2 (1.5)	40 (29.4)	
Confidence in the service			0.02
Yes	38 (27.9)	70 (51.5)	
No	4 (2.9)	24 (17.6)	
Explanations as to health status?			0.55
Yes	39 (29.7)	88 (64.7)	
No	3 (2.2)	6 (4.4)	
Did team show politeness and respect?			0.10
Yes	42 (30.9)	88 (64.7)	
No	0 (0)	6 (4.4)	
Opinion as to the model of care			0.00
Adequate	17 (12.5)	14 (10.2)	
Inadequate	25 (18.4)	80 (58.8)	
Do you know the name of the professional who cared for you?			0.43
Yes	30 (22.1)	70 (51.5)	
No	12 (8.8)	24 (17.6)	
Concept of risk (need for care)			0.46
Dangerous situation	14 (10.3)	29 (21.3)	
Severe disease, death	28 (20.6)	65 (47.8)	
Before going to the ER, were you seen at the primary care?			0.30
Yes	13 (9.6)	23 (17.0)	
No	29 (21.3)	71 (52.2)	
Reason for seeking the ER			0.01
Acute complaint	15 (11.0)	53 (39.0)	
Chronic complaint	6 (4.4)		
Medical care	21 (15.4)		

*c^2^ (p<0.05). ER: emergency room.

The cleanliness and comfort of the facilities were significantly associated (p≤0.05) with satisfaction/dissatisfaction (Table 2).

**Table 2:** Resultado da análise bivariada entre a satisfação/insatisfação e as variáveis relativas ao ambiente (n=136)

Variables	Satisfied n (%)	Dissatisfied n (%)	p value*
Cleanliness of the facilities			0.00
Adequate	33 (24.3)	45 (33.1)	
Inadequate	9 (6.6)	49 (36.0)	
Comfort of the facilities			0.00
Adequate	21 (15.4)	15 (11.0)	
Inadequate	21 (15.4)	79 (58.1)	
Quality of the facilities			0.06
Adequate	33 (24.4)	60 (44.4)	
Inadequate	9 (6.7)	33 (24.4)	
In the ER, do you know where to complain?			0.52
Yes	13 (9.6)	28 (20.6)	
No	29 (21.3)	66 (48.5)	
Did you have to pay for any service?			0.18
Yes	18 (13.2)	31 (22.8)	
No	24 (17.6)	63 (46.3)	

*c^2^ (p<0.05). ER: emergency room.

## DISCUSSION

The quality of management and care may be known by means of user satisfaction evaluations, and by their expectations and needs regarding the services rendered, in a necessary process that provides useful information.^12,16^

As to the delay in care and waiting time, we perceived that a significant number of clients showed dissatisfaction, a result similar to that of other studies.^2,6,17,18)^ However, in a study done at a large urgent care center in San Diego, Venezuela, the patients demonstrated satisfaction with this aspect.^19^A systematic review showed that the reason for greatest dissatisfaction among the patients is waiting time. Easy admission and simple and complete reception are the initial point for avoiding user dissatisfaction.^1^ Admission and reception are essential elements for quality of care, as they allow one to effectively act on the individual’s health and that of the community, and can favor the reorganization of the services and qualification of the care given.^20^

As to confidence of the user in the service, which proved to be associated with dissatisfaction, we point out that the users bring with them their individuality represented by their beliefs and values. Therefore, is it considered that, from the user’s point of view, the professional-patient relation should be based on attention, care, friendship, competence, warmth, and skills, besides good dialogue between the parts, in which the professional respects the fragility of the user, in order to contribute, as well, to quality care.^21,22^ A population-based cross-sectional study performed in Porto Alegre (RS) ratified such a premise, since it identified that the fact of having been treated well by the physician during the clinical visit was directly related to greater patient satisfaction.^18^

The opinion on an inadequate model of care proved to be significantly associated with dissatisfaction with care delivered. A study carried out at the university hospital of São Carlos (SP), which evaluated the satisfaction of users, showed that dissatisfaction of patients hinders both their treatment and the organization rendering care, which is therefore co-responsible for excellence or failure of the care given. The relation between service providers and their users is formed, basically, by bonds formed between the quality of service offered and satisfaction of users that receive care. An appropriate functional model of care becomes high user satisfaction. Some actions of the users, such as compliance to treatment, continuation with the long-term care, search for care that can promote health, and recommendation of the service to others are associated with this satisfaction.^23^

The present study showed that most of the clientele sought help due to an acute complaint and that a large proportion reported dissatisfaction with care received. It was also noted that most of the considerable number (33%) of users who were at the urgent care service for a medical consultation were also dissatisfied. These data confirm a reality: the user emphasizes the cure and not health promotion, prevention of problems, and initial care, which confirms the dependence on the hospital-centered model. Inversely, they should have chosen first primary care instead of hospital services, especially in case of chronic complaints or routine medical care, thus reducing waiting time and lack of beds, and contributing to expediting services.^23^

In face of this panorama in the Brazilian cities that daily deal with the difficult problem of overcrowding, lack of patient risk classification, and absence of integration between primary care and tertiary care, it is necessary to adopt strategies and measures such as those observed in studies conducted in Ohio^24^ and in Chicago,^25^ in the United States, as well as in Germany.^26^ Such studies showed many opportunities for hospitals to improve their emergency rooms and urgent care services, primarily by means of continuity of patient care and integration among different levels, as well as through professional training, interaction, and collective participation among department managers, hospital directors, and healthcare professionals – all fully involved in true improvement of the urgent care and emergency services.^24^

It is worth mentioning the experience of the Pediatric Hospital of Michigan, in the United States, which was able to reduce admissions by 83% at the urgent care center and the length of stay by 48%, by eliminating the waiting lines. At this hospital, initially, specific data were sought as to quality of care, creating an efficiency operation. There was alignment of the existing routes in order to improve the interpretations of morbidity and mortality data, using the opportunities of medical visits, transfers of care, and rules and regulations. This was possible because of the use of individual or small group simulation models, to predict the intervention pathways before changes.^8^ However, there are differences that can be attributed to diversities among Brazil, United States and European countries, notably due to better income distribution, and well-established healthcare systems in the latter countries.^18^

In this investigation, dissatisfaction also kept an association with the judgment of inadequate cleanliness and comfort of the facilities, similar to the study carried out at a university hospital in Rio Grande do Sul (RS), in which the patients demonstrated feeling uncomfortable with aspects relative to hospital facilities, such as infrastructure (lack of beds and noise).^6^A study that thoroughly evaluated the urgent care and emergency services of the hospital network in the Northeastern part of Brazil^5^ and another done at organizations in Wiesbaden and in the district of Rheingau-Taunus, in Germany,^26^ also corroborated these findings, since they identified difficulties in infrastructure of the environment and material resources. In the German study, this deficiency generated dissatisfaction even among the healthcare professionals.^26^ This unsatisfactory result may also be explained by the lack of physical structure for the patients awaiting care. Many are admitted to the corridor until they can obtain a vacancy in one of the beds of the emergency room.^6^ Nevertheless, in a study done in an emergency room of a teaching hospital in the state of Paraná (PR),^12^ and in another private hospital in the city of São Paulo (SP),^16^ the clients revealed that they were satisfied with the cleanliness and comfort of the facilities.

Infrastructure is one of the factors considered most relevant for patient satisfaction, even more than the cure itself. The facilities evaluation includes cleanliness, equipment, appropriate furniture, and sufficient ventilation, so that appropriate care provides a dignified, welcoming, and comfortable and effective admission.^23^

In Brazil, the Ministry of Health foresees the need for improving healthcare, and for this it uses various strategies, such as the Policy of Qualification of Healthcare at the SUS, created to elevate the level of quality of care delivered to the population by the public health services, leading to greater satisfaction with the system and to legitimation of the healthcare policy developed in Brazil. The National Humanization Policy has the proposal of establishing a Network of Humanization in Healthcare to promote reduction of waiting lines and waiting time, in addition to the implementing risk classification at triage.^27^ There is also the National Survey of SUS User Satisfaction at different levels of care, with the object of studying satisfaction and perception of the user as one of the evaluation components of the system.^13^ It is expected that these public policies will be truly applied in order to establish improvement in care given within the context of emergency rooms and urgent care centers.

The study had limitations: the cross-sectional design, which hinders declarations of cause and effect, and the restriction to the local scenario, making possible generalizations difficult.

## CONCLUSION

The study showed that there was great dissatisfaction on the part of the clients, aggravated by the associated factors of inadequate waiting time, lack of confidence in the service, inappropriate model of care, reason for seeking help related to an acute complaint, and inadequate cleanliness and comfort of the facilities. These results indicate aspects that require improvements and indicate a critical attitude of patients. It is believed that they may be applied as guides in improvements of quality of care and satisfaction rates. It is important to point out that the factors that compromised the satisfaction of the patients, along with the lack of integration among the levels of care were characterized as hindrances for the quality of urgent care and emergency care. Healthcare managers and professionals should increasingly include the client in the process of evaluation of care, aiming to implement strategies that revert the situation identified and promote care with truly good quality.
