# Supplementary material for: Quality of emergency rooms and urgent care services: user satisfaction
Source: Einstein (Sao Paulo). 2015 Oct-Dec;13(4):587–93. doi: 10.1590/S1679-45082015GS3347 (PMC4878635; doi:10.1590/S1679-45082015GS3347)
Supplement: Translation

# Translation: Qualidade dos prontos-socorros e prontos-atendimentos: a satisfação dos usuários

**Authors:** Cássio de Almeida Lima, Bruna Tatiane Prates dos Santos, Dina Luciana Batista Andrade, Francielle Alves Barbosa, Fernanda Marques da Costa, Jair Almeida Carneiro

**Affiliations:** 1 Universidade Estadual de Montes Claros, Montes Claros, MG, Brasil.; 2 Faculdade de Saúde Ibituruna, Montes Claros, MG, Brasil.

**Keywords:** Satisfação do paciente, Assistência ao paciente, Qualidade da assistência à saúde, Avaliação em saúde, Serviços médicos de emergência, Questionários

## Abstract

**Objetivo:**

Avaliar a qualidade dos prontos-socorros e prontos atendimentos, de acordo com a satisfação dos usuários desses serviços.

**Métodos:**

Estudo descritivo transversal com abordagem quantitativa. A amostra foi constituída aleatoriamente por 136 usuários. A coleta de dados ocorreu no período de outubro e novembro de 2012 por meio de questionário estruturado.

**Resultados:**

Os participantes eram na maioria do sexo masculino (64,7%), com idade inferior aos 30 anos (55,8%) e a escolaridade predominante foi o ensino médio (54,4%). Entre os itens avaliados, os que se associaram estatisticamente com os níveis de satisfação com o atendimento foram: tempo de espera, confiança do serviço, modelo do atendimento e motivo da procura do atendimento relacionado à queixa aguda, limpeza e conforto do ambiente.

**Conclusão:**

A acessibilidade, o acolhimento e a infraestrutura foram fatores considerados mais relevantes para a satisfação do paciente do que a cura propriamente dita.

## INTRODUÇÃO

O fenômeno mundial da superlotação que ocorre nos serviços de emergência hospitalar é também uma realidade brasileira. Vários fatores estão envolvidos na gênese desse problema; entre eles, os mais expressivos são o aumento da violência urbana, a maior incidência dos agravos de causa externa e o envelhecimento populacional, que influencia no aumento da prevalência de doenças crônicas degenerativas. Essa demanda aumentada por serviços hospitalares de caráter emergencial implica em demora no atendimento e na sobrecarga da equipe de saúde, gerando uma assistência de baixa qualidade.^(1,2)^

A dificuldade do serviço de urgência e emergência na saúde vem desde a Atenção Primária à Saúde (APS), devido à escassez de profissionais especializados, em paralelo com a baixa resolutividade do atendimento e as reduzidas estratégias de promoção da saúde, levando o usuário a procurar o serviço de pronto-socorro, pois este se encontra aberto 24 horas. Desse modo, o pronto-socorro recebe uma alta demanda, que interfere nos cuidados prestados à clientela que necessita do autêntico cuidado emergencial, o que prejudica também os procedentes da APS, obrigando o cliente passar por recusas, longas filas de espera, morosidades e demora de atendimentos.^(3,4)^

Não obstante os processos em evolução no Sistema Único de Saúde (SUS), os princípios constitucionais devem ser respeitados e perseguidos. Da mesma forma, os diversos aspectos comprometedores da qualidade da assistência hospitalar, como os relacionados aos recursos humanos e à organização das Redes de Atenção à Saúde, as quais devem ser integradas, merecem ser objeto de avaliação e efetivas intervenções.^(5)^ Nesse cenário, assume notável relevância a avaliação da assistência na visão do usuário, ou seja, sua satisfação quanto à qualidade dessa assistência. No Brasil, as pesquisas de satisfação dos usuários receberam destaque a partir da década de 1990, em função da redemocratização do país e do surgimento de movimentos de luta pelos direitos sociais, especialmente os de acesso aos serviços de saúde.^(2)^

De tal modo, propicia-se ao usuário a oportunidade de opinar sobre as políticas públicas e identificar os fatores determinantes de sua satisfação, em sintonia com a recomendação da Organização Mundial da Saúde (OMS) de que os gestores considerem as expectativas dos cidadãos na tomada de decisão. Para tanto, a satisfação do paciente tem sido adotada pelas instituições de saúde como estratégia para obter um conjunto de percepções relacionado à qualidade da atenção recebida, com o qual se adquire informações que beneficiam a organização dos serviços.^(6-9)^

A partir de então, a literatura se tornou bastante ampla nesse assunto. Contudo, são necessários estudos mais específicos, que apontem aspectos diretamente relacionados à qualidade do serviço e à satisfação dos usuários, com destaque para os fatores relacionados ao cliente, aos profissionais de saúde e à qualidade das instalações.^(10-13) ^Uma vez que o alvo da assistência é o paciente, nada mais justo do que buscar a qualidade em suas expectativas, com vistas à sua satisfação como um aspecto determinante no julgamento da qualidade.

Nesse sentido, em busca de maior qualidade dos cuidados de saúde, em que se destacam os serviços de urgência e emergência, observa-se um crescente aumento na quantidade de avaliações pelas instituições, em nível nacional e internacional. Isso porque pesquisar a satisfação do paciente oportuniza sua participação em um processo de avaliação e tomada de decisão, que promove melhorias efetivas e adequadas à clientela assistida, o que, por sua vez, está em consonância com as recomendações atuais da OMS e com os pressupostos de participação social do SUS. Assim, acredita-se que este estudo deve garantir informações úteis aos gestores e profissionais que prestam a assistência nos serviços de urgência e emergência, além de subsidiar mais discussões a respeito do assunto.

## OBJETIVO

Avaliar a qualidade dos prontos-socorros e prontos atendimentos de acordo com a satisfação dos usuários desses serviços.

## MÉTODOS

Tratou-se de estudo descritivo, de cunho transversal e abordagem quantitativa. Para a efetivação da pesquisa, foram incluídos os hospitais da cidade de Montes Claros (MG): Hospital Universitário Clemente de Faria (HUCF), Fundação Hospitalar de Montes Claros – Hospital Aroldo Tourinho (HAT), Irmandade Nossa Senhora das Mercês de Montes Claros (Santa Casa) e Fundação de Saúde Dilson de Quadros Godinho (FDQG).

O foco do estudo foram os prontos-socorros e prontos-atendimentos desses hospitais que representaram o universo hospitalar da cidade deMonte Carlos (MG). Ressalta-se que todos esses serviços eram porta de entrada para os usuários, não sendo exigido referenciamento para ser atendido. Vale ressaltar algumas particularidades dos serviços, a saber: somente o HUCF era um pronto-socorro que atendia exclusivamente pacientes do SUS. Nesse pronto-socorro, havia capacidade instalada para 36 leitos de observação ou serviço de atendimento intermediário para aqueles que aguardavam internação. Para cada plantão de 12 horas, o serviço contava com um médico clínico geral, um pediatra, um cirurgião e um ortopedista, além de dois enfermeiros, sendo um responsável pela triagem e outro pela assistência; contava ainda com seis técnicos de enfermagem. Quanto aos demais serviços, estes atendiam a pacientes do SUS, de convênios e particulares.

HAT, Santa Casa e HUCF dispunham de pronto-socorro com atendimento nas 24 horas. A FDQG dispunha apenas de pronto atendimento. No pronto-socorro do HAT havia 25 leitos; em cada plantão de 12 horas, havia um médico clínico geral, um pediatra, um cirurgião, um ortopedista e um neurologista, além de dois enfermeiros e quatro técnicos de enfermagem. Na Santa Casa, o pronto-socorro possuía 40 leitos e contava com a seguinte equipe: dois médicos clínicos, dois pediatras, um cirurgião, um ortopedista, um cardiologista e um neurologista, além de dois enfermeiros e oito técnicos de enfermagem. Quanto à FDQG, no pronto atendimento havia 12 leitos, além de uma equipe plantonista composta por um médico clínico geral, um pediatra, um cirurgião, dois enfermeiros e três técnicos de enfermagem.

Para definição do tamanho da amostra, utilizou-se o corte de 20% de todos os sujeitos atendidos em um dia de atendimento normal do serviço. A base para efetivação do cálculo foi o número de atendimentos diários registrado por meio do sistema de informação ambulatorial, ou seja, utilizou-se a média diária de atendimentos. À época da coleta dos dados, os serviços atendiam diariamente as seguintes quantidades médias de pacientes: HUCF − 100 pacientes; HAT − 100 pacientes; Santa Casa − 300 pacientes; FDQG − 180 pacientes. O total foi de 680 pacientes/dia em média.

Considerando 20% do total, a amostra foi composta por 136 usuários. A opção por 20% da população justificou-se pelo fato de se tratar de uma população infinita e flutuante, para a qual deveria ser definida uma proporção, que poderia variar entre 10 e 50%. Contudo, por se tratar de um fenômeno corriqueiro e recorrente − atendimento em serviços de saúde − a proporção de 20% era suficiente para definir e caracterizar o fenômeno.^(14)^

Foi considerada a amostragem aleatória e estratificada. Os extratos do processo de amostragem foram de cada um dos prontos-socorros ou prontos-atendimentos. Considerando que cada serviço de saúde, em estudo, era um extrato, foram respeitadas as devidas proporções do número de atendimento/dia de cada serviço, sendo investigadas diferentes quantidades de usuários em cada serviço em relação ao total de 136 pacientes/dia em prontos-socorros e prontos-atendimentos em Montes Claros (MG), que ficaram assim definidos: HUCF com 14,7% (20 indivíduos); HAT com 14,7% (20 indivíduos); Santa Casa com 44,1% (60 indivíduos) e FDQG com 26,4% (36 indivíduos).

A seleção dos participantes do estudo ocorreu por meio de sorteio a partir das listas de pessoas que tiveram o risco classificado pelo protocolo de *Manchester *e que estavam em espera de retorno e liberação após atendimento médico. Foram incluídos nos estudo os usuários que já tinham sido atendidos e aguardavam ser liberados e que não apresentavam, no momento da entrevista, condição limitante para a participação no estudo, como dor intensa ou algum outro desconforto significativo; apresentavam idade superior a 18 anos; e aceitaram responder ao questionário mediante a prévia leitura e assinatura do Termo de Consentimento Livre e Esclarecido.

A coleta de dados foi realizada entre outubro e novembro de 2012, por meio de um questionário aplicado aos usuários selecionados. Durante a aplicação dos questionários, os clientes que eram chamados para o atendimento tiveram a entrevista interrompida, para não atrasar o atendimento; estes foram automaticamente eliminados da amostra. Nesse caso, o participante era aleatoriamente substituído por outro. A aplicação dos questionários aconteceu sempre no período diurno.

O questionário utilizado para a coleta de dados era estruturado e validado pelo Ministério da Saúde, por meio do Programa Nacional de Avaliação de Serviços Hospitalares.^(15)^ Foram incluídas outras questões acerca do conhecimento dos usuários sobre o que era atendimento de urgência e emergência, e sobre como a rede assistencial deveria ser organizada.

As variáveis independentes do estudo foram agrupadas em sociodemográficas, características do atendimento do serviço de urgência e emergência; e características do ambiente. Variáveis sociodemográficas corresponderam a idade, sexo e escolaridade. As variáveis características do atendimento do serviço de urgência e emergência foram tempo de espera; confiança no serviço; se recebeu esclarecimento sobre o estado de saúde; respeito da equipe médica e de enfermagem; e forma de atendimento, além de nome do profissional que o atendeu, conceito de risco de seu próprio quadro de saúde, se antes de ir ao pronto-socorro procurou algum serviço de APS, motivo da procura pelo serviço. As variáveis características do ambiente foram adequação da limpeza, qualidade e conforto do ambiente, se o usuário sabia onde reclamar em caso de insatisfação. A variável dependente foi definida pela seguinte pergunta: “Você está satisfeito com o serviço que recebeu no pronto-socorro ou pronto-atendimento?” Na categoria satisfeito, foram incluídos os que referiram estar muito satisfeitos e satisfeitos, enquanto os insatisfeitos ou muito insatisfeitos foram incluídos na categoria insatisfeitos.

As análises estatísticas, foram realizadas utilizando o *softwareStatistical Package for Social Sciences *(SPSS), versão 18.0 para *Windows*. Após análise descritiva, a associação entre o relato de satisfação com o atendimento e as variáveis independentes foi investigada por meio de análise bivariada, empregando o teste χ^2^ de *Pearson*. Para todas as análises, foi considerado um valor de p≤0,05.

O projeto de pesquisa foi aprovado pelo Comitê de Ética em Pesquisa das Faculdades Unidas do Norte de Minas da Associação Educativa do Brasil, com parecer 225.868/2013 e número CAAE: 06316712.1.0000.5141.

## RESULTADOS

Participaram do estudo 136 usuários dos serviços de urgência e emergência. Os resultados mostraram grande insatisfação com o atendimento dos prontos-socorros e prontos-atendimentos dos hospitais em estudo: 94 (69,1%) desses usuários estavam insatisfeitos. A amostra foi composta por pacientes, que eram, em sua maioria, do sexo feminino (88; 64,7%), com idade de até 30 anos (76; 55,9%) e a escolaridade predominante foi o ensino médio (74; 54,4%).

Os fatores associados à satisfação e à insatisfação na análise bivariada (p≤0,05) foram: demora no atendimento, tempo de espera, confiança no serviço, opinião sobre o modelo de atendimento e motivo de procura pelo pronto-socorro (Tabela 1).

**Tabela 1:** Resultado da análise bivariada entre a satisfação/insatisfação, e as variáveis relativas ao perfil do usuário e características do atendimento (n=136)

Variáveis	Satisfeito n (%)	Insatisfeito n (%)	Valor p*
Sexo			
Feminino	16 (11,8)	32 (23,5)	0,40
Masculino	26 (19,1)	62 (45,5)	
Idade			
Menos de 30 anos	22 (16,2)	54 (39,7)	0,35
Mais de 30 anos	20 (14,7)	40 (29,4)	
Escolaridade			
Ensino superior	7 (5,1)	12 (8,8)	0,35
Ensino médio	19 (14,0)	55 (40,4)	
Analfabeto até o ensino médio	16 (11,8)	27 (20,0)	
Características do atendimento			
Demora no atendimento			0,00
Não demorou	6 (4,4)	0 (0)	
Demorou	36 (26,5)	94 (69,1)	
Tempo de espera			0,00
Até 120 minutos	40 (29,4)	54 (39,7)	
Mais de 120 minutos	2 (1,5)	40 (29,4)	
Confiança no serviço			0,02
Sim	38 (27,9)	70 (51,5)	
Não	4 (2,9)	24 (17,6)	
Esclarecimento sobre estado de saúde			0,55
Sim	39 (29,7)	88 (64,7)	
Não	3 (2,2)	6 (4,4)	
Equipe demonstrou educação e respeito?			0,10
Sim	42 (30,9)	88 (64,7)	
Não	0 (0)	6 (4,4)	
Opinião sobre o modelo de atendimento?			0,00
Adequado	17 (12,5)	14 (10,2)	
Inadequado	25 (18,4)	80 (58,8)	
Sabe o nome do profissional que o atendeu			0,43
Sim	30 (22,1)	70 (51,5)	
Não	12 (8,8)	24 (17,6)	
Conceito de risco (necessidade de atendimento)			0,46
Situação perigosa	14 (10,3)	29 (21,3)	
Doença grave, morte	28 (20,6)	65 (47,8)	
Antes de ir ao PS foi atendido na APS?			0,30
Sim	13 (9,6)	23 (17,0)	
Não	29 (21,3)	71 (52,2)	
Motivo da procura pelo PS			0,01
Queixa aguda	15 (11,0)	53 (39,0)	
Queixa crônica	6 (4,4)		
Atendimento médico	21 (15,4)		

*χ^2^ (p<0,05). PS: pronto-socorro; APS: atenção primária à saúde.

A limpeza do ambiente e o conforto se associaram significativamente (p≤0,05) à satisfação/insatisfação (Tabela 2).

**Tabela 2:** Resultado da análise bivariada entre a satisfação/insatisfação e as variáveis relativas ao ambiente (n=136)

Variáveis	Satisfeito n (%)	Insatisfeito n (%)	Valor p*
Limpeza do ambiente			0,00
Adequada	33 (24,3)	45 (33,1)	
Inadequada	9 (6,6)	49 (36,0)	
Conforto do ambiente			0,00
Adequada	21 (15,4)	15 (11,0)	
Inadequada	21 (15,4)	79 (58,1)	
Qualidade do ambiente			0,06
Adequada	33 (24,4)	60 (44,4)	
Inadequada	9 (6,7)	33 (24,4)	
No PS sabe onde pode reclamar?			0,52
Sim	13 (9,6)	28 (20,6)	
Não	29 (21,3)	66 (48,5)	
Teve que pagar por algum serviço?			0,18
Sim	18 (13,2)	31 (22,8)	
Não	24 (17,6)	63 (46,3)	

*c^2^ (p<0,05). PS: pronto-socorro.

## DISCUSSÃO

A qualidade da gestão e da assistência pode ser conhecida por meio de avaliações da satisfação dos usuários, e das suas expectativas e necessidades sobre o serviço prestado, num processo necessário que fornece informações úteis.^(12,16)^

Com relação à demora do atendimento e ao tempo de espera, percebe-se que quantidade significativa dos clientes mostrou-se insatisfeita, resultado similar ao de outros estudos.^(2,6,17,18) ^Porém, em pesquisa feita em um serviço de urgência em San Diego, na Venezuela, os pacientes se mostraram satisfeitos com esse aspecto.^(19) ^Revisão sistemática evidenciou que o motivo de maior insatisfação entre os pacientes é o tempo de espera. O fácil acesso e o acolhimento simples e completo, são, assim, o ponto inicial para evitar a insatisfação do usuário que utiliza o serviço.^(1)^ O acesso e o acolhimento são elementos essenciais para a qualidade do atendimento, permitem atuar efetivamente sobre a saúde do indivíduo e da coletividade, e podem favorecer a reorganização dos serviços e a qualificação da assistência prestada.^(20)^

Quanto à confiança do usuário no serviço, que se mostrou associada à insatisfação, ressalta-se que os usuários trazem consigo sua individualidade representada por suas crenças e valores. Logo, é considerado que, na visão do usuário, a relação profissional-paciente deve-se fundamentar em atenção, cuidado, amizade, competência, carinho e habilidade, além de um bom diálogo entre os mesmos, em que o profissional respeite a fragilidade do usuário, a fim de contribuir, também, para uma assistência de qualidade.^(21,22)^ Estudo transversal de base populacional realizado em Porto Alegre (RS) ratifica tal premissa, pois foi identificado que o fato de ter sido bem tratado pelo médico durante a consulta esteve diretamente relacionado com uma maior satisfação.^(18)^

A opinião de um modelo de atendimento inadequado se mostrou associada significativamente à insatisfação com a assistência. Uma pesquisa feita no hospital-escola de São Carlos (SP), que avaliou a satisfação dos usuários, mostrou que a insatisfação do paciente prejudica tanto seu tratamento quanto a instituição prestadora do cuidado, sendo ela, assim, corresponsável pela excelência ou pelo fracasso da assistência prestada. A inter-relação entre as prestadoras de serviços e seus usuários é formada, basicamente, pela vinculação entre a qualidade do serviço oferecido e a satisfação do usuário que o recebe. O modelo de funcionamento adequado do serviço converte-se em alta satisfação do usuário. Ações dos usuários, como aderência ao tratamento, prosseguimento dos cuidados em longo prazo, busca por cuidados que podem promover a saúde e recomendação do serviço a outros estão associados a essa satisfação.^(23)^

O presente estudo evidenciou que a maior parte da clientela procurou por queixa aguda e que, desta, grande parte relatou insatisfação com o atendimento. Observou-se, ainda, que a maioria do número considerável (33%) de usuários que estavam no serviço de urgência para consulta médica também estava insatisfeita. Estes dados comprovam uma realidade: o usuário dá ênfase à cura e não à promoção da saúde, à prevenção de agravos e aos cuidados iniciais, o que confirma a dependência do modelo hospitalocêntrico. Inversamente, deveriam optar primeiramente pela APS, ao invés de recorrer ao serviço hospitalar, principalmente diante de queixas crônicas ou atendimentos médicos rotineiros, reduzindo o tempo de espera e a falta de leitos, e contribuindo para a agilidade no atendimento.^(23)^

Diante desse panorama nas cidades brasileiras, que lidam no dia a dia com o difícil entrave da superlotação, da falta de classificação de risco dos pacientes e da ausência da integração entre a APS e os setores de nível terciário, é preciso adotar estratégias e medidas como as observadas em estudos feitos em Ohio^(24)^ e em Chicago,^(25)^ nos Estados Unidos, e na Alemanha.^(26)^ Tais estudos evidenciaram amplas oportunidades para os hospitais melhorarem seus serviços de prontos-socorros e prontos-atendimentos, principalmente por meio da continuidade da assistência ao paciente e a integração entre diferentes serviços de níveis diversos, assim como por meio da capacitação profissional, da interação e da participação coletiva entre os gestores de setor, diretores do hospital e profissionais da assistência − todos com envolvimento na melhoria real dos serviços de urgência e emergência.^(24-26)^

É interessante citar a experiência do Hospital Pediátrico de Michigan, nos Estados Unidos, que conseguiu reduzir a admissão em 83% no pronto-atendimento e o tempo de permanência no local em 48%, com a eliminação das filas de espera. Nesse hospital, inicialmente, foram buscados dados específicos sobre a qualidade no atendimento, criando-se uma operação de eficiência. Houve um alinhamento das vias existentes, para melhorar as interpretações de dados sobre morbidade e mortalidade, utilizando-se oportunidades de consultas, transferências de cuidados e regras de regulamento. Isso foi possível graças à utilização dos modelos de simulação individual ou em pequenos grupos, para predizer as vias de intervenção antes das mudanças.^(8)^ Contudo, há diferenças que podem ser atribuídas às diversidades entre o Brasil, Estados Unidos e países europeus, notadamente devido à melhor distribuição de renda e aos sistemas de saúde bem estruturados nesses últimos.^(18)^

Nessa investigação, a insatisfação também se manteve associada ao julgamento de limpeza e conforto inadequados, similarmente ao estudo feito em hospital universitário no Rio Grande do Sul (RS), no qual os pacientes demonstraram incômodo com aspectos relativos ao ambiente hospitalar como infraestrutura (falta de leitos e barulho).^(6)^ Pesquisa que avaliou amplamente os serviços de urgência e emergência da rede hospitalar de referência no Nordeste brasileiro^(5)^ e outra feita em instituições de Wiesbaden e no distrito de Rheingau-Taunus, na Alemanha,^(26)^ também corroboram esses achados, uma vez que foram constatadas dificuldades de infraestrutura do ambiente e recursos materiais. No estudo alemão, essa deficiência gerou insatisfação até mesmo nos profissionais de saúde.^(26)^ Esse resultado insatisfatório ainda pode ser explicado pela falta de estrutura física para os pacientes aguardarem o atendimento. Muitos ficam internados no corredor até obterem uma vaga nos leitos do pronto-socorro.^(6)^ No entanto, em pesquisa no pronto-socorro de um hospital de ensino do Paraná (PR),^(12)^ e em outra em hospital privado da cidade de São Paulo (SP),^(16)^ os clientes revelaram estarem satisfeitos com a limpeza e o conforto do ambiente.

A infraestrutura é um dos fatores considerados mais relevantes para a satisfação do paciente, do que propriamente sua cura. O ambiente deve abranger limpeza, equipamentos, mobílias apropriadas e ventilação suficiente, para que um atendimento apropriado efetive uma atenção com acesso digno, acolhedor e confortável.^(23)^

No Brasil, o Ministério da Saúde prevê a necessidade de se melhorar a assistência à saúde, diante da qual utiliza várias estratégias, como a Política de Qualificação da Atenção à Saúde no SUS, criada para elevar o nível de qualidade da assistência à saúde prestada à população pelo SUS, levando a uma maior satisfação com o sistema e à legitimação da política de saúde desenvolvida no Brasil. A Política Nacional de Humanização tem como proposta a constituição da Rede de Humanização em Saúde, a fim de promover a redução das filas e dos tempos de espera, além da implantação do acolhimento com classificação de risco.^(27)^ Há ainda a Pesquisa Nacional de Avaliação da Satisfação dos Usuários do SUS nos diferentes níveis de atendimento, tendo como objeto de estudo a satisfação e a percepção do usuário como um dos componentes da avaliação do sistema.^(13)^ Espera-se que essas políticas públicas sejam realmente aplicadas a fim de concretizar a melhoria do cuidado prestado no contexto dos prontos-socorros e prontos-atendimentos.

O estudo teve limitações: o desenho transversal, que impede declarações de causa e efeito, e a restrição ao cenário local, dificultando-se possíveis generalizações.

## CONCLUSÃO

O estudo evidenciou que houve grande insatisfação por parte dos clientes, agravada pelos fatores associados: tempo de espera inadequado, falta de confiança no serviço, modelo de atendimento inapropriado, motivo da procura relacionado à queixa aguda, limpeza e conforto inapropriados. Esses resultados indicam aspectos que exigem melhorias e sinalizam uma postura crítica dos pacientes. Acredita-se que eles podem ser aplicados como norteadores de melhorias na qualidade do atendimento e nos índices de satisfação. Vale ressaltar que os fatores que comprometeram a satisfação dos pacientes, aliados à falta de integração entre os níveis de Atenção à Saúde, caracterizam-se como entraves para a qualidade dos serviços de urgência e emergência. Os gestores e profissionais de saúde devem incluir, cada vez mais, o cliente no processo de avaliação dos cuidados, almejando a efetivação de estratégias que revertam a situação constatada e que promovam uma assistência de real qualidade.
